# A systematic review and meta‐analysis of investigating the mutual impact of COVID‐19 and psoriasis: Focusing on COVID‐19 course in psoriasis and the opinion on biologics in this setting

**DOI:** 10.1002/iid3.1063

**Published:** 2023-11-07

**Authors:** Nazila Ghoreishi Amin, Sepehr Khosravi, Najmolsadat Atefi, Farnoosh Seirafianpour, Sahand Farhoodi, Azadeh Goodarzi

**Affiliations:** ^1^ Department of Radiology, Keck School of Medicine University of Southern California (USC) Los Angeles California USA; ^2^ Non‐Communicable Diseases Research Center, Endocrinology and Metabolism Population Sciences Institute Tehran University of Medical Sciences Tehran Iran; ^3^ Department of Dermatology, Rasool Akram Medical Complex Clinical Research Development Center (RCRDC) Iran University of Medical Sciences Tehran Iran; ^4^ Razi Drug Research Center Iran University of Medical Sciences Tehran Iran; ^5^ School of Medicine Iran University of Medical Sciences Tehran Iran

**Keywords:** biologics, COVID‐19, cytokine storm, dermatology, flare‐up, hyperinflammation, immune‐mediated diseases, immunosuppressive treatments, inflammation, nonbiologics, psoriasis, systematic review

## Abstract

**Introduction:**

This systematic review and meta‐analysis aims to investigate the mutual impact of COVID‐19 and psoriasis to inform clinical practice and future research.

**Methods:**

We followed the Preferred Reporting Items for Systematic Reviews and Meta‐analysis protocol for systematic reviews and searched PubMed, Web of Science, Scopus, and Google Scholar until May 1, 2022. Eligibility criteria included full‐text articles in English reporting COVID‐19 treatment outcomes in psoriasis patients. Studies on animals, letters to editors, non‐English studies, and studies with no access to full articles were excluded. Search results were screened and data were extracted by two groups of reviewers with any discrepancies resolved by the senior author. The risk of bias was assessed using ROBINS‐I for nonrandomized studies. The hospitalization rate, Intensive Care Unit (ICU) admission rate, case fatality rate, odds ratios of COVID‐19 infection and hospitalization rate in psoriasis patients were extracted and analyzed using random effects analysis to calculate pooled prevalence and odds ratios, as well as to explore heterogeneity.

**Results:**

We found 1980 records from four databases and included 20 studies after screening and removing duplicates. These studies evaluated 185,000 psoriasis patients and included eight retrospective cohort studies, one case‐control study, three cross‐sectional studies, and eight case series studies. The impact of the COVID‐19 pandemic on psoriasis treatment and the outcome of COVID‐19 infection in psoriasis patients receiving different forms of treatment were evaluated. The pooled data from included studies showed that the incidence rate of COVID‐19 infection among psoriasis patients was 0.03% (confidence interval [CI]: 0.01–0.06), with a pooled odds ratio of 1.97 (CI: 0.69–5.60) compared to the general population. The hospitalization rate, ICU admission rate, and case fatality rate for psoriasis patients with COVID‐19 were 0.17 (CI: 0.10–0.31), 0.06 (CI: 0.06–0.46), and 0.02 (CI: 0.01–0.04), respectively. Additionally, psoriasis patients receiving systemic nonbiologic therapy had a pooled odds ratio of 2.32 (CI: 1.18–4.57) for hospitalization compared to those using biologic agents.

**Conclusion:**

Studies have shown that biologic therapy for psoriasis did not increase the risk of hospitalization due to COVID‐19 infection and may have even offered some protection. Treatment adherence was higher in psoriasis patients receiving biologic therapies than those receiving conventional therapies. These findings suggest that psoriasis treatment did not negatively impact COVID‐19 infection and that treatment could be continued on a case‐by‐case basis during the pandemic.

## Highlights

- The use of immunosuppressive treatments in patients with pre‐existing immune‐mediated diseases, such as psoriasis, who become infected with COVID‐19 is a controversial issue.
- COVID‐19 can cause cytokine storm in the body, which may exacerbate diseases with inflammation‐based pathophysiology.
- Current information about the dynamic association between COVID‐19 and pre‐existing immune‐mediated diseases, as well as the impact of COVID‐19 infection and its treatments on the course of the underlying disease, is limited and controversial.
- There are concerns that discontinuing or altering routine medications due to COVID‐19 may change the course of the underlying disease, and flare‐ups may occur.
- However, evidence suggests that biologics and nonbiologic drugs may have desirable effects on overcoming the hyperinflammation state caused by COVID‐19, and withholding biologics due to COVID‐19 infection is not advisable.
- Patients who had discontinued their psoriasis medications prior or after being afflicted with COVID‐19 were more likely to experience severe psoriasis flare‐ups.
- Patients who had not missed their scheduled biologic administration were likely to experience mild COVID‐19 symptoms while their psoriasis condition remained unchanged.
- Using glucocorticoids is one of the main therapeutic strategies in patients with severe COVID‐19 symptoms, but in psoriatic patients, administration of glucocorticoids during the course of COVID‐19 infection might affect the patient's psoriasis presentation. It is crucial to further investigate this matter, as there is still no general agreement.
- Infection with COVID‐19 does not increase the possibility of exacerbation and flare‐up of psoriasis.

## 1 INTRODUCTION

The COVID‐19 pandemic has significantly impacted the world's health systems, with over 300 million confirmed cases and 5 million deaths reported globally as of March 2023.^1^ One of the major concerns during the pandemic is how pre‐existing medical conditions may impact the severity and outcomes of COVID‐19, particularly for those with chronic inflammatory diseases such as psoriasis and how COVID‐19 could impact the treatment of psoriasis.^2^ From the early phases of the pandemic until now, studies on the impact of COVID‐19 on patients with psoriasis have been ongoing. The findings of studies conducted during the early pandemic differ from those of more recent studies, and there are still lessons to be learned from these studies. Initially, it was thought that patients with psoriasis and those receiving immunosuppressive treatment were at higher risk of severe outcomes from COVID‐19 infection compared to the general population. However, studies on this matter reported otherwise.^2^, ^3^, ^4^, ^5^, ^6^, ^7^, ^8^ There were discussions on the continuation and discontinuation of systemic biologic therapy in psoriasis patients, and studies on this matter also reported various findings.^9^, ^10^, ^11^ Given the different and sometimes contradictory results reported by studies on the impact of COVID‐19 on psoriasis patients, a comprehensive systematic review is needed to synthesize the available evidence and better understand the relationship between COVID‐19 and psoriasis. This review will provide valuable insights into the potential implications for clinical practice and future research and inform the development of guidelines for managing patients with psoriasis. The aim of this study is to systematically review, analysis and investigate the mutual impact of COVID‐19 and psoriasis on each other.

## 2 METHODS

Our protocol included the Preferred Reporting Items for Systematic Reviews and Meta‐analysis (PRISMA) protocol for the systematic reviews.^12^, ^13^ An electronic search with English language restriction was conducted in PubMed, Web of Science, Scopus, and Google Scholar until May 1, 2022.

### 2.1 Eligibility criteria

Full‐text articles with English language restriction reporting COVID‐19 treatment outcomes in psoriasis patients or the impact of COVID‐19 on the treatment of psoriasis are included. Studies on animals, letters to editors, non‐English studies, and studies with no access to full articles were excluded from this study.

### 2.2 Data sources and search strategy

We performed a search strategy for PubMed, Scopus, Web of Science, and Google Scholar until May 1, 2022. Citations are all included studies were also evaluated to ensure all studies that met inclusion criteria were included. The search strategy of this study is available in the Supplement file.

### 2.3 Study selection and data extraction

Search results were imported into EndNote (X9, Thomson Reuters), duplicates were removed, and two groups of two reviewers screened titled and abstracts independently. Any reviewer discrepancies were resolved in a session with the senior author. After obtaining full texts, two reviewers checked them with inclusion and exclusion criteria. Again, in this step, the senior author resolved any disagreements between the two reviewers. Finally, the included studies were reviewed for data extraction after reviewing the complete text. We extracted the first author, publication year, study location, type of study, number of patients, pathology of the patients, study description, and conclusion. For the meta‐analysis, other variables such as patients' treatment regimen for psoriasis, type of medication used, number of male and female patients, publication year, country, COVID‐19 infection rate, hospitalization rate, Intensive Care Unit (ICU) admission rate, case fatality rate, comorbidities such as respiratory, cardiovascular, diabetes, and hypertension were also extracted.

### 2.4 Quality assessment

For risk of bias assessment, the RoB2 tool was used for randomized clinical trials and ROBINS‐I for nonrandomized studies as recommended by Cochrane Handbook for Systematic Reviews of Intervention.^14^, ^15^, ^16^

### 2.5 Statistical analysis

Incidence of COVID‐19 infection, hospitalization rate, ICU admission rate, and case fatality rate of psoriasis patients were extracted from all included studies. Pooled prevalence and corresponding 95% confidence interval (CIs) were calculated with random effects analysis. The odds ratio (OR) of COVID‐19 infection in psoriasis patients to the general population and OR of hospitalization rate in psoriasis patients receiving systemic nonbiologic therapy to patients on a biologic agent were extracted from the studies. Pooled OR was calculated with random effects analysis. Further exploration for the source of heterogeneity was conducted in case of significant heterogeneity. Other information, such as comorbidities like respiratory, cardiovascular, and diabetes, were extracted from all included studies. Due to the number of included studies (<10), the meta‐regression analysis was unreliable for reporting. Subgroup analysis as recommended by Cochrane Handbook for Systematic Reviews of Intervention^14^, ^15^, ^16^ was chosen for further exploration was chosen and the subgroup analysis of countries is reported. All statistical meta‐analysis were conducted with R software, version 4.2.3 to generate forest plots, pooled OR, and pooled prevalence with 95% CIs.

## 3 RESULTS

**Figure 1:**
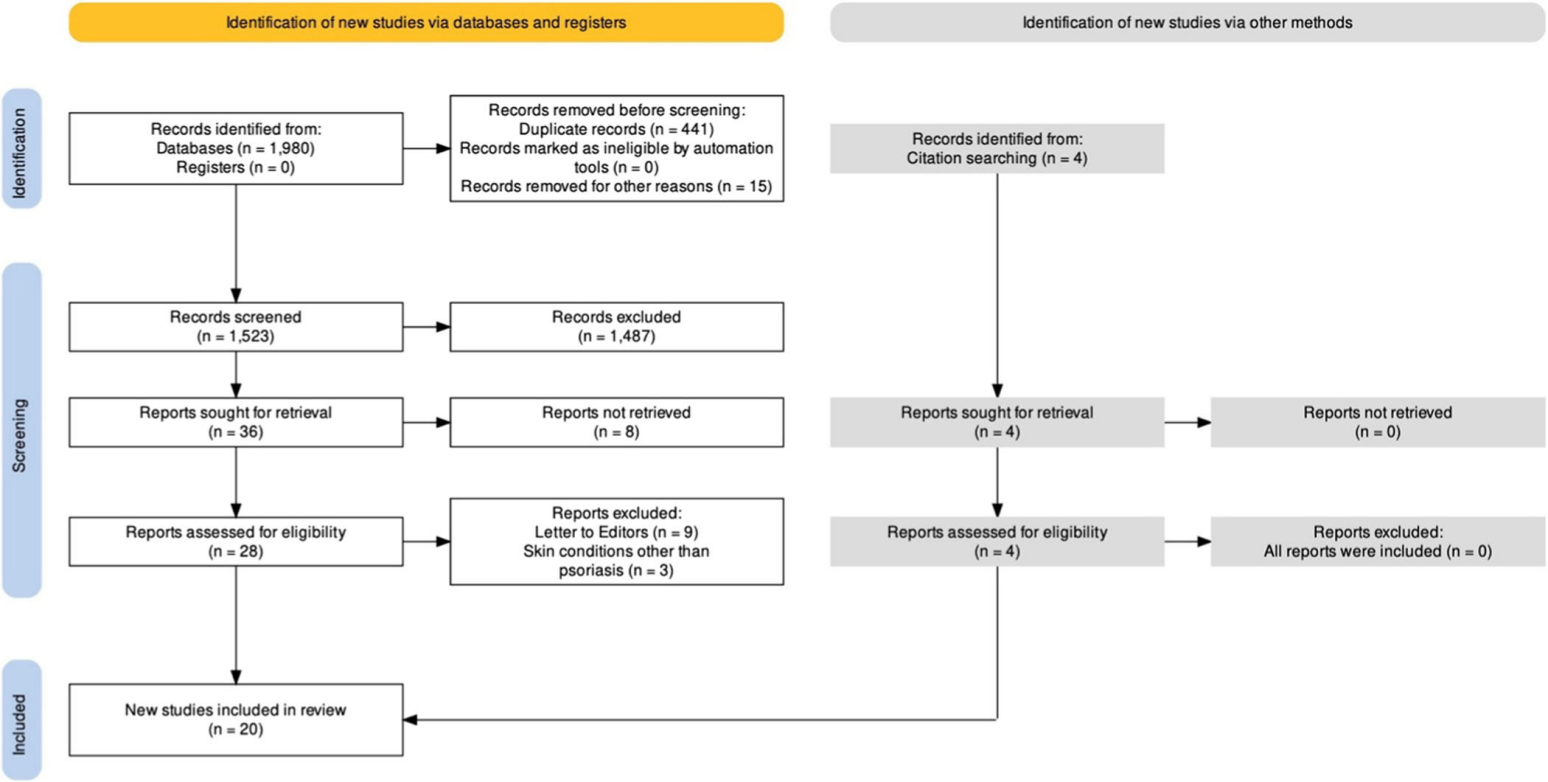
Preferred Reporting Items for Systematic Reviews and Meta‐analysis flowchart of included studies.

**Figure 2:**
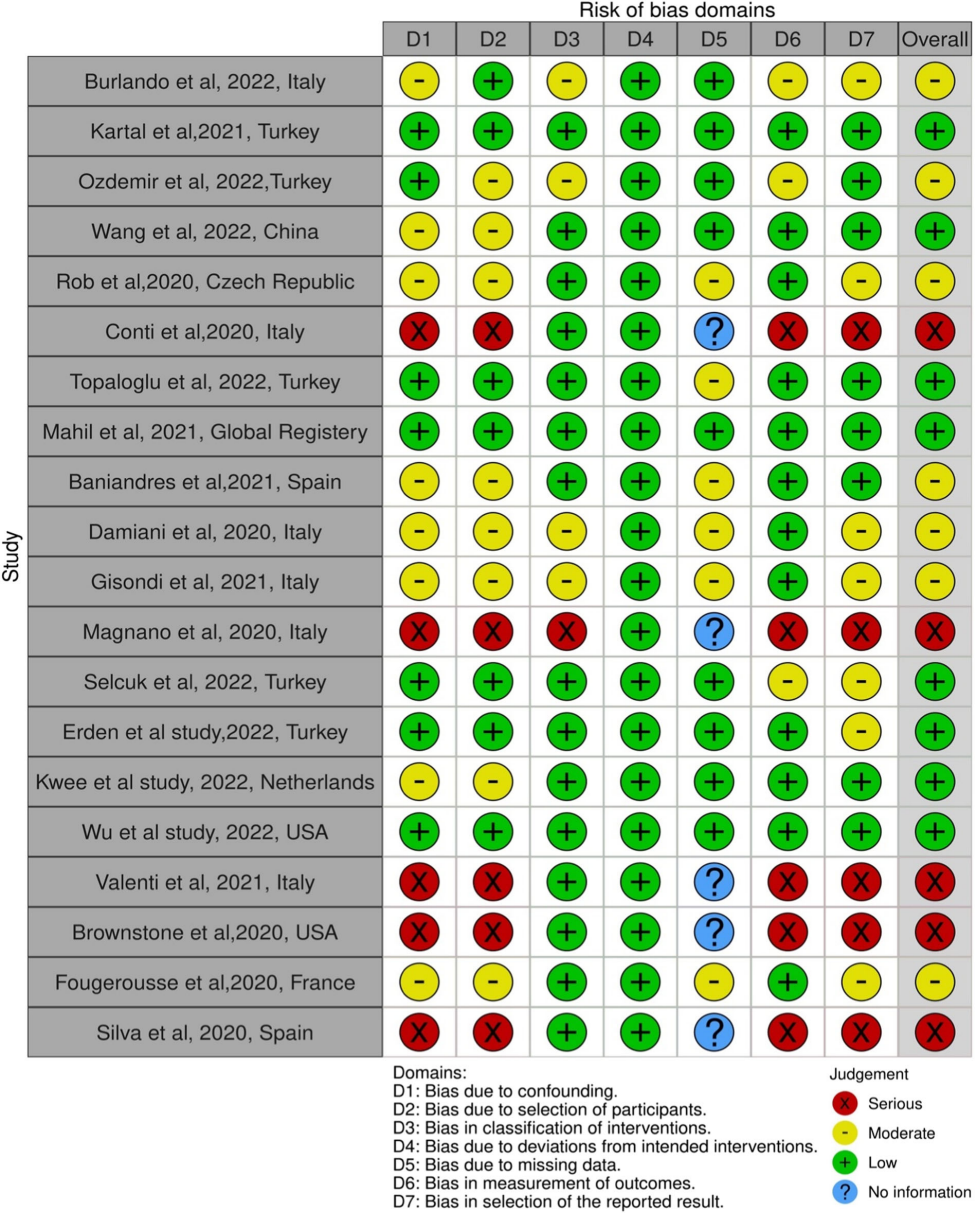
Risk of bias assessment of included studies.

In total, 1980 records from four databases (Scopus, Web of Science, PubMed, and Google Scholar) were found with our selection criteria. After removing duplicate records, 1523 studies were screened, 16 studies were included, and four other studies were included by evaluation of citations of included studies accounting for a total of 20 studies. The PRISMA flowchart of this study is presented in Figure 1. In total, 185,000 psoriasis patients were evaluated in the included studies. Out of 20 studies, eight studies were retrospective cohort studies, one case–control study, three cross‐sectional studies, and eight were case series studies. The risk of bias assessment of included studies results is presented in Figure 2. Because of the unprecedented situation of the COVID‐19 pandemic, all the included studies were nonrandomized studies and ROBINS‐I was used to assess the risk of bias in all studies. According to the ROBINS‐I guideline, eight studies evaluated as low risk, seven as moderate risk, and five as a serious risk for bias. COVID‐19 pandemic impacted the treatment of psoriasis patients and studies evaluated the impact of the pandemic on the continuation and discontinuation of different forms of psoriasis treatment (nonbiologic systemic therapy, biologic systemic therapy, and topical therapy) which is presented in Table 1. Also, patients with psoriasis who receive different forms of treatment were infected with COVID‐19 infection and the outcome of the COVID‐19 infection in these patients was evaluated in studies (presented in Table 2).

**Table 1:** COVID‐19 pandemic effects on psoriasis treatment.

Study	Study design	Number of patients	Pathology	Study description	Outcome
Burlando et al.,^17^ Italy	Case series	515	Psoriasis and psoriatic arthritis	Survey from 515 Psoriasis and psoriatic arthritis patients	None were hospitalized. Out of 27 patients who discontinued their biologic in nine patients, psoriasis worsened from PASI 90 to PASI 75. A total of 488 patients who continued their biologic treatment, no patient reported hospitalization due to COVID‐19 or worsening of psoriasis.
Kartal et al.,^7^ Turkey	Cross sectional	1827	Psoriasis	Psoriasis patients receiving immunosuppressive treatment and recording drug adherence and effect discontinuation on disease worsening	The drug adherence rate was 68.2%, with patients receiving anti‐IL drugs more likely to continue treatment than those receiving conventional drugs. The disease worsening rate was 24.2%, with drug dose reduction and drug withdrawal increasing this rate. Receiving anti‐TNF or anti‐IL drugs was associated with less disease worsening compared to conventional drugs. Four patients had mild COVID‐19 infection with an incidence of 0.0022%, while the incidence in the general population was 0.0025%.
Ozdemir et al.,^10^ Turkey	Retrospective cohort	246	Psoriasis	Dermatology clinic recorded psoriasis patients' admission for receiving treatment and compare the data in three phases of prepandemic, early pandemic, and late pandemic	Psoriasis patients who were receiving biologics adherence to treatment affected drastically and the highest treatment interruption rate were in patients receiving biologic agents.
Wang et al.,^18^ China	Cross sectional	926	Psoriasis	A survey on 926 psoriasis patients about adherence to psoriasis treatment during COVID‐19 pandemic	A total of 68.5% of patients did not adhere to psoriasis treatment and the treatment adhere was lower in patients with systematic and topical treatment compared to biological treatment. Nonadherence to treatment was associated with the aggravation of psoriasis in a significant number of patients.
Rob et al.,^19^ Czech Republic	Retrospective cohort	210	Psoriasis	In total, 210 psoriasis patients were included in this study for further investigation on adherence to treatment of psoriasis	None of the patients receiving biologic treatment discontinued their treatment but the anxiety and safety concerns in groups receiving biologic treatment were significantly higher than other psoriasis patients.
Conti et al.,^20^ Italy	Case series	4	Psoriasis	Four patients with psoriasis receiving biologics who had contact risk of infection with COVID‐19	Treatment was discontinued in two asymptomatic patients and no sign of worsening of psoriasis were reported.
Topaloglu et al.,^21^ Turkey	Retrospective cohort	169	Psoriasis	Moderate and severe psoriasis patients (measured by PASI) receiving biologics and discontinued their treatment due to COVID‐19 pandemic	Clinical worsening (change of PASI score) reported in 41.4% and relapse in 48.5% of total patients. The significant risk factor for clinical worsening and relapse were alcohol use during biologic‐free period, total time of biologics, and presence of additional triggering factors. Also, use of secukinumab and ustekinumab were found to be protective of clinical worsening.

Abbreviation: IL, interleukins; PASI, Psoriasis Area and Severity Index; TNF, tumor necrosis factor.

**Table 2:** Effect of psoriasis treatment on COVID‐19 infection.

Study	Study design	Number of patients	Pathology	Study description	Outcome
Mahil et al.,^22^ Global Registery	Case series	347	Plaque psoriasis, mild psoriasis, and psoriatic arthritis	Patients with psoriasis and COVID‐19 infection were registered and the outcome of COVID‐19 infection treatment is recorded. Also, variables associated with COVID‐19 infection complications in patients with psoriasis were reported.	Psoriasis patients who were receiving biologic therapy compared to those receiving nonbiologic systemic therapy had lower rates of hospitalization, mechanical ventilation, and death. Of all psoriasis patients, 93% fully recovered from COVID‐19 infection. No significant difference was found in risk of hospitalization between different classes of biological agents.
Baniandres et al.,^3^ Spain	Retrospective cohort	2329	Psoriasis	Analysis of incidence of COVID‐19 infections and its severe outcomes in a cohort of psoriatic patients treated with systemic therapies.	SIR for COVID‐19 infection, hospitalization, ICU care, and death were higher compared to general population but this result was not statistically significant.
Conti et al.,^20^ Italy	Case series	4	Psoriasis	Four patients with psoriasis receiving biologics who had contact risk of infection with COVID‐19.	Out of four patients, only two patients showed symptoms of COVID‐19 infection and the other two showed no sign of infection.
Damiani et al.,^23^ Italy	Case control	1193	Psoriasis	A total of 1193 psoriasis patients treated with biologics and small molecules were compared to general population of Lombardia, Italy.	Patients treated with biologics were at a higher risk of being infected with COVID‐19 compared to general population but they were not at increased risk of ICU admission.
Gisondi et al.,^24^ Italy	Retrospective cohort	6501	Chronic plaque psoriasis	Recording incidence and hospitalization rate COVID‐19 infection in 6501 patients and comparing the rates to general population for comparison. In total, 980 psoriasis patients were infected with COVID‐19.	There was no significant difference between incidence and hospitalization of psoriasis patients on treatment compared with general population.
Magnano et al.,^25^ Italy	Case series	9	Psoriasis	Out of a pool of 720 psoriasis patients, nine had confirmed COVID‐19 infection swab test. All of the patients were receiving biologic agents and their COVID‐19 infection were followed up.	In eight out of nine patients, the physicians decided to withdraw biologic therapy to avoid other microbial infections. Only one patient with obesity, hypertension, diabetes, and chronic renal failure were hospitalized and was admitted to the ICU but recovered.
Selcuk et al.,^9^ Turkey	Retrospective cohort	488	Psoriasis	A total of 488 psoriasis patients were evaluated and rare of hospitalization due to COVID‐19 infection among them and general population were reported. Rate of hospitalization among biological and nonbiological treatment of psoriasis were also evaluated.	Patients receiving biological treatment and nonbiological treatment for psoriasis did not differ significantly in hospitalization rate due to COVID‐19 infection except for the ones receiving acitretin.
Erden et al.,^4^ Turkey	Retrospective cohort	1216	Psoriasis	The mortality rate of psoriasis patients before and after COVID‐19 pandemic were compared to each other and to general population.	A twofold increase of crude mortality reported comparing pre‐ and during pandemic mortality of psoriasis patients. Comparing to general population mortality rate of psoriasis patients does not have significant difference to general population both before and during pandemic.
Kwee et al.,^8^ The Netherlands	Cross‐sectional study	551	Psoriasis	Evaluated risk of COVID‐19 infection in psoriasis patients and compare the risk of infection in patients receiving biologic and or nonbiologic systemic therapy to those treated with topical treatment.	Psoriasis patients receiving biologic and nonbiologic treatment did not have increased risk of COVID‐19 infection and hospitalization compared to those receiving topical treatment. Only four patients in total were hospitalized and none of the patients were admitted to the ICU.
Wu et al.,^11^ The United States	Retrospective cohort	167,027	Psoriasis	Patients' data with psoriasis diagnosis collected from Symphony Health data set and infection with COVID‐19 and their psoriasis treatment were analyzed.	Patients with psoriasis have 18% higher odds of COVID‐19 infection incident compared to general public (OR = 1.18). Psoriasis patients receiving no systematic therapy were at higher risk of hospitalization due to COVID‐19 infection compared with biologic treatment. Patients using TNF inhibitor, methotrexate, and apremilast had decreased odds of COVID‐19 incident compared to topical therapy.
Valenti et al.,^26^ Italy	Case report	1	Psoriasis and psoriatic arthritis	Patient with COVID‐19 infection was treated with Adalimumab for psoriasis, simultaneously.	Simultaneous treatment with Adalimumab did not cause COVID‐19 respiratory distress or other complications of COVID‐19 infection.
Brownstone et al.,^27^ The United States	Case series	2	Psoriasis	Two cases with psoriasis who were infected with COVID‐19 infection.	Successful recovery of both patients and neither of them were hospitalized.
Fougerousse et al.,^5^ France	Cohort	1418	Psoriasis	Adult psoriasis patients receiving systemic or biologic treatment.	From all the patients, 0.35% needed hospitalization and 60% of whom had another risk factor for severe COVID‐19 infection.
Silva et al.,^28^ Spain	Case series	7	Psoriasis and psoriatic arthritis	Comparison of psoriasis patient receiving biologic immunosuppressant therapy versus apremilast.	Patients receiving apremilast compared to patients receiving Infliximab presented with less severe form of COVID‐19 infection.
Kartal et al.,^7^ Turkey	Cross sectional	1827	Psoriasis	Psoriasis patients receiving immunosuppressive treatment and recording drug adherence and effect discontinuation on disease worsening.	The study concluded that psoriasis patients using systemic immunosuppressive do not have a higher COVID‐19 risk than the general population and treatment compliance with biological drugs is higher.

Abbreviations: ICU, Intensive Care Unit; OR, odds ratio; SIR, Standardized Incidence Rate.

**Figure 3:**
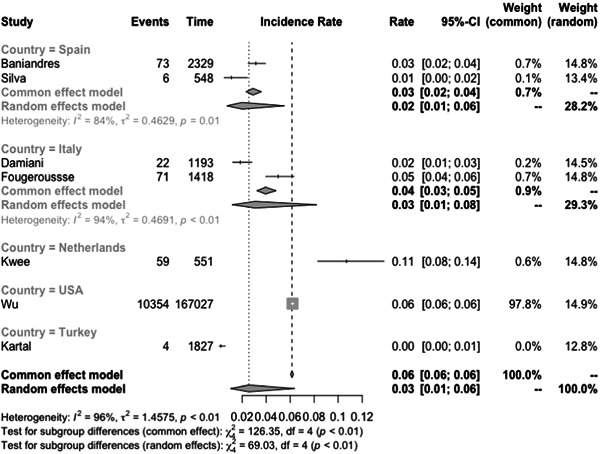
Incidence of COVID‐19 infection in psoriasis patients.

**Figure 4:**
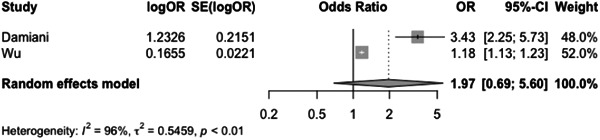
Odd ratio of COVID‐19 infection in psoriasis patients compared to the general population.

**Figure 5:**
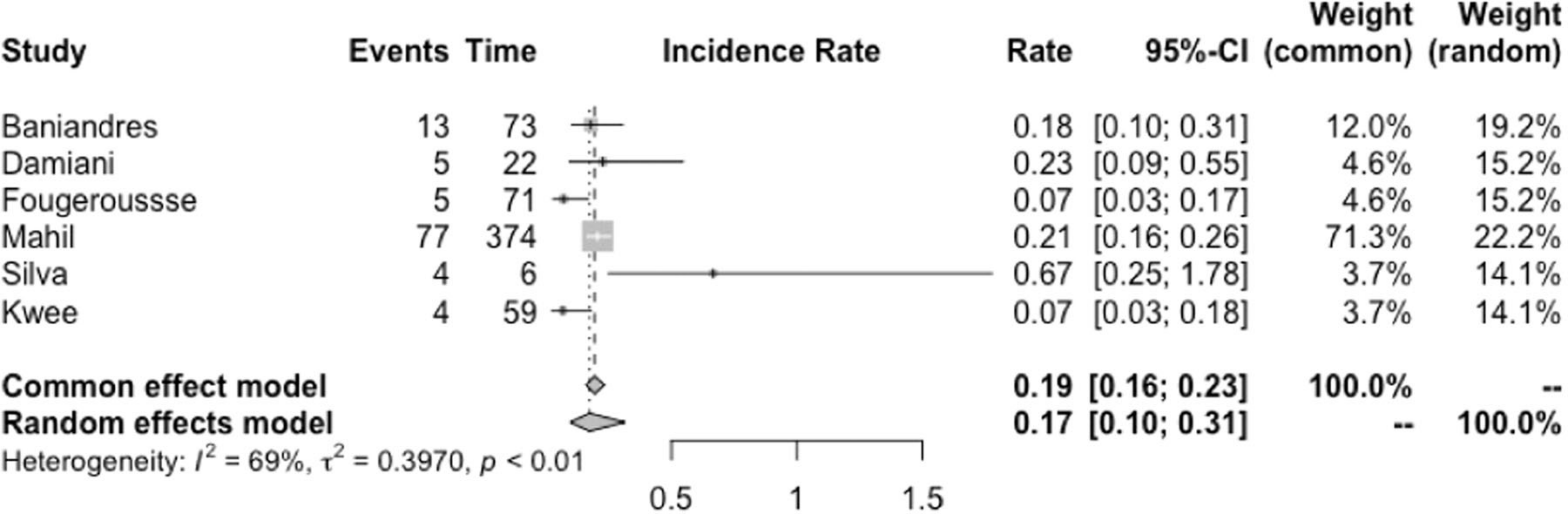
Hospitalization rate of psoriasis patients infected with COVID‐19.

**Figure 6:**
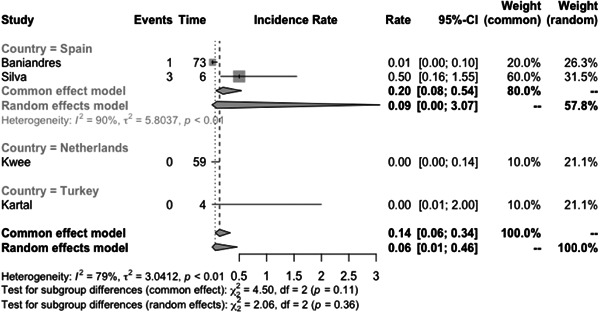
Intensive Care Unit admission rate of psoriasis patients infected with COVID‐19.

**Figure 7:**
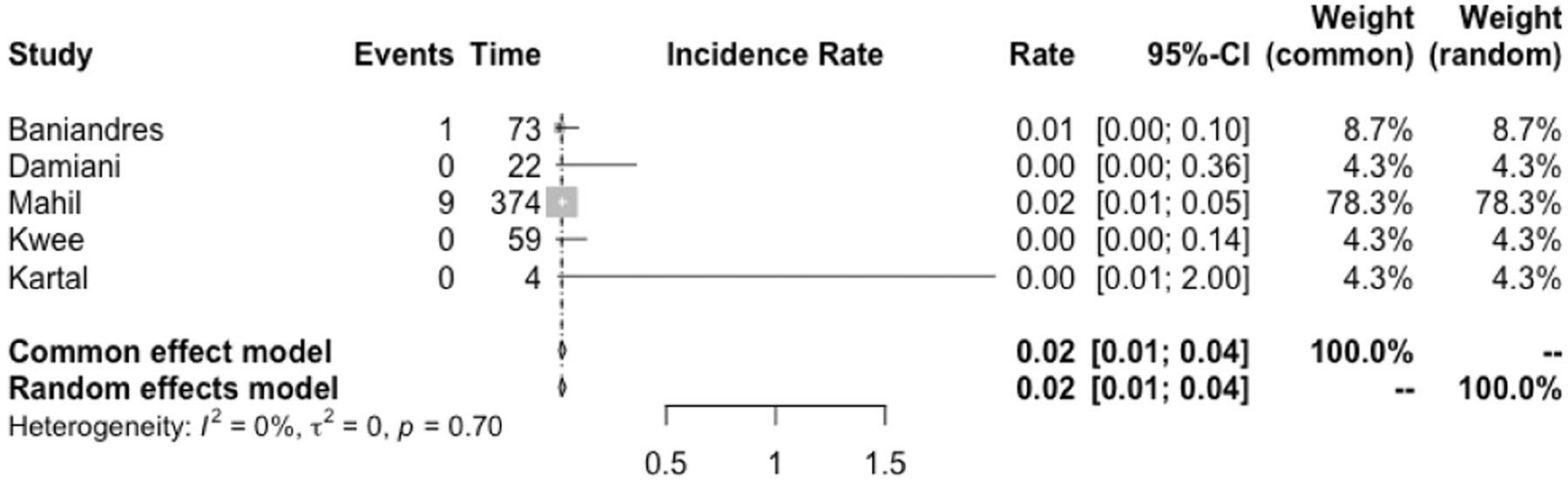
Case fatality rate of psoriasis patients infected with COVID‐19.

**Figure 8:**
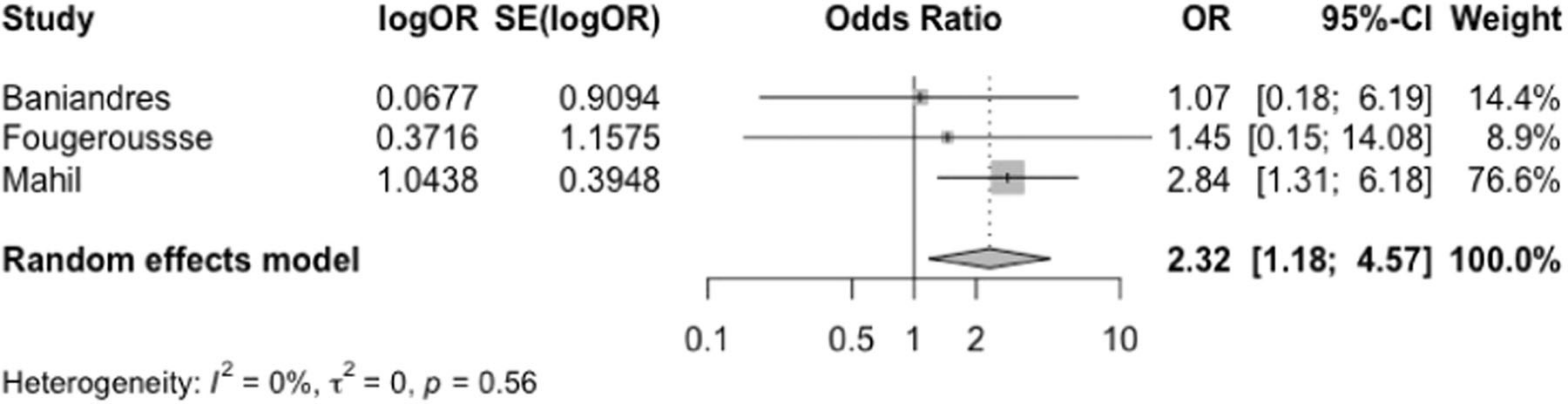
Odd ratio of hospitalization in psoriasis patients receiving systemic nonbiologic therapy to use of a biologic.

The incidence rate of COVID‐19 infection among psoriasis patients was determined to be 0.03% (CI: 0.01–0.06) based on the pooled data from the included studies (Figure 3). The pooled OR of COVID‐19 infection in psoriasis patients compared to the general population was found to be 1.97 (CI:0.69–5.60) (Figure 4). The hospitalization rate, ICU admission rate, and case fatality rate of psoriasis patients infected with COVID‐19 were calculated to be 0.17 (CI: 0.10–0.31), 0.06 (CI: 0.06–0.46), and 0.02 (CI: 0.01–0.04), respectively (Figures 5, 6, and 7). Furthermore, a pooled OR of 2.32 (CI: 1.18–4.57) was observed for hospitalization among psoriasis patients receiving systemic nonbiologic therapy compared to those using biologic agents (Figure 8).

## 4 DISCUSSION

The COVID‐19 pandemic, officially declared on March 11, 2020, has had an unprecedented impact on healthcare over the past 3 years.^29^ Studies examining the relationship between psoriasis patients and COVID‐19 have yielded varied and sometimes contradictory findings. The mutual impact of COVID‐19 infection and psoriasis warrants further investigation. A review of the evidence accumulated over the past 3 years could provide valuable insights for the medical community. Psoriasis patients typically receive various forms of treatment, including systemic biologic therapy, nonbiologic systemic therapy, and topical therapy which could impact COVID‐19 infection in them. Also, COVID‐19 pandemic has disrupted the treatment of many psoriasis patients. The literature reports on the impact of treatment continuation or discontinuation on COVID‐19 infection among psoriasis patients.

A review of current studies on psoriasis patients receiving immunosuppressive treatment shows various findings. Several studies reported that psoriasis patients are not at higher risk of hospitalization and severe infection compared to general population but studies also reported that these patients have higher risk of infection with COVID‐19 compared to the general population.^3^, ^4^, ^6^, ^7^, ^20^, ^23^, ^24^, ^25^, ^30^, ^31^ Our study found a pooled OR of 1.97 (CI: 0.69–5.60) for the incidence of COVID‐19 infection in psoriasis patients compared to the general population. This result is consistent with the aforementioned finding, which further supports our conclusion. The observed OR suggests that psoriasis patients may be at a slightly increased risk of contracting COVID‐19. Also, our analysis of published studies, through a pooled prevalence approach, revealed a prevalence rate of COVID‐19 infection among psoriasis patients of 0.03 (0.01–0.06). Baniandres et al.^3^ examined the incidence of COVID‐19 infections and severe outcomes among psoriatic patients receiving systemic therapies and compared it to the general population. Among 2329 patients receiving systemic therapy, 73 (3.13%) contracted COVID‐19, 13 (0.56%) were hospitalized, one (0.04%) required ICU care, and one (0.04%) died. The standardized incidence ratios (SIRs) for COVID‐19 infection, hospitalization, ICU care, and death were slightly higher among psoriatic patients receiving systemic therapies than in Spain's general population. Still, the difference was not statistically significant.^3^ Our analysis of pooled hospitalization rate, ICU admission rate, and case fatality rate among psoriasis patients infected with COVID‐19 revealed a hospitalization rate of 0.17 (CI: 0.10–0.31), an ICU admission rate of 0.06 (CI: 0.01–0.46), and a case fatality rate of 0.02 (CI: 0.01–0.04). Findings on hospitalization rate, ICU admission rate, and case fatality rate of COVID‐19 in general population is different between countries but the reported hospitalization rate in the literature is not significantly higher than psoriasis patients.^32^ This finding is further confirmed by the findings of Erden et al.,^4^ which reported a twofold increase of crude mortality reported comparing pre‐ and during‐pandemic mortality of psoriasis patients while compared to the general population mortality rate of psoriasis patients does not have a significant difference to the general population both before and during the pandemic.

Our meta‐analysis aimed to compare hospitalization rates among psoriasis patients receiving systemic nonbiologic therapy versus biologic agent therapy. Our results indicate that patients treated with nonbiologic systemic therapy have a higher risk of hospitalization compared to those on biologic agents (OR: 2.32 [1.18–4.57]). This finding is consistent with previous studies in the literature, which have shown that nonbiologic systemic therapy is associated with a higher risk of hospitalization compared to biologics.^7^ In a retrospective multicenter cohort study by Gisondi et al.^24^ of 6501 patients with chronic plaque psoriasis treated with biologic therapy in Northern Italy, the incidence rate of hospitalization for COVID‐19 was found to be 11.7 (95% CI, 7.2–‐18.1) per 10,000 person‐months in patients with psoriasis and 14.4 (95% CI, 14.3–14.5) in the general population.^24^ The SIR of hospitalization and death in patients with psoriasis compared with the general population was 0.94 (95% CI, 0.57–1.45; *p* = .82) and 0.42 (95% CI, 0.07–1.38; *p* = .19), respectively. The study found no significant difference in hospitalization rates with the general population when stratifying by age or by class of biologic and suggests the continuation of biologic therapies. Further studies also compared systemic biologic and nonbiologic systemic therapy regarding severe COVID‐19 infection outcomes. Kartal et al. study data did not show a negative impact of immunosuppressive on the COVID‐19 infection course in psoriasis patients, and none of the COVID‐19‐positive cases required hospitalization. Increased hospitalization risk was associated with older age, male sex, nonwhite ethnicity, and comorbid conditions.

Studies also compared different biologics to each other in terms of COVID‐19‐related risks. Data from the Psoriasis Longitudinal Assessment and Registry showed that rates of severe infection were not more remarkable for ustekinumab or etanercept but were higher for adalimumab and infliximab compared to patients on nonbiologic/nonmethotrexate psoriasis therapy.^31^ This contradicts a prospective study from the Association of British Dermatologists Biologic Intervention Register, which found no significantly higher risk of severe infections for etanercept, adalimumab, and ustekinumab compared with nonbiologic therapies for psoriasis patients.^33^ Biologic treatment using immunosuppressive drugs such as guselkumab, ustekinumab, adalimumab, secukinumab, brodalumabor ixekizumab may even protect against the onset and evolution of COVID‐19 infection.^20^, ^34^, ^35^ It is worth mentioning that more recent studies also reported a protective effect of hospitalization in biologics users. For instance, Wu et al. study reported that contrary to previous reports, biologics agents have a protective effect in preventing hospitalization from COVID‐19 infection, and biologic agents like TNF inhibitors, methotrexate, and apremilast decreased the incidence of COVID‐19 infection.^11^, ^36^ This finding about the protective effect of apremilast is supported by more studies^28^ and reported as a safe choice among biologics in psoriasis patients infected with COVID‐19 due to its immunomodulatory properties and specific mechanism of action.^37^, ^38^ In Selcuk et al. study,^9^ patients receiving biological and nonbiological treatment for psoriasis did not significantly differ in hospitalization rate due to COVID‐19 infection except for those receiving acitretin. Findings on the protective effect of biologics were contrasting, but an emphasis on the protective effect of apremilast is found in the literature.

The COVID‐19 pandemic has also impacted the treatment of psoriasis. For various reasons, some patients have discontinued their medicine, either with or without consulting a dermatologist. At the onset of the pandemic, there was disagreement among dermatologists on the continuation or initiation of biologic drugs due to a lack of reliable evidence on the course of COVID‐19 in psoriatic patients and the effect of biologics on the disease.^39^, ^40^ Additionally, patients' attitudes toward receiving treatment changed in this period because of difficulties accessing healthcare (quarantine and isolations) or concerns about the safety of these drugs.^41^, ^42^

A study by Kartal et al. included a large population of 1827 psoriasis patients receiving conventional immunosuppressive (methotrexate and cyclosporine) and systemic biologic therapy (anti‐TNF or anti‐IL drugs).^7^ According to the study, most patients (68.2%) continued their treatment during the pandemic. Treatment adherence was higher in psoriasis patients receiving biologic therapies than conventional therapies, with the anti‐IL‐using group having the highest treatment adherence. This result agrees with previous reports stating that conventional systemic treatments and topical therapy were associated with worse adherence than systemic biologic therapy.^18^, ^19^, ^43^, ^44^, ^45^, ^46^, ^47^ However, few studies have contrary findings on adherence to treatment, specific adherence to biologic agents. Ozdemir et study. Reported the interruption of treatment follow‐ups was pronounced among patients receiving biologics, with only 8.5% returning for follow‐up compared to 18.1% of patients receiving other treatments (nonbiologic systemic therapy and topical therapy).^10^

Studies also reported that discontinuing psoriasis treatment in some cases has resulted in the worsening of psoriasis. Topaloglu et al. study reported^21^ clinical worsening (change of PASI score) reported in 41.4% and relapse in 48.5% of total patients. The significant risk factor for clinical worsening and relapse were alcohol use during the biologic‐free period, total time of biologics, and the presence of additional triggering factors. Also, using secukinumab and ustekinumab were protective of clinical worsening.^21^, ^44^ Wang et al. study reported that during the COVID‐19 epidemic, nonadherence to treatment was associated with the aggravation of psoriasis conditions, perceived stress, and symptoms of anxiety and depression.^18^ Strategies targeting adherence to treatment, including telemedicine, health education, and drug supplies, are necessary for patients with psoriasis, in addition to mental health interventions.^48^, ^49^, ^50^ The study conducted by Luigi Gargiulo and colleagues presented a case series involving 28 individuals who either developed new‐onset psoriasis or experienced exacerbations of their pre‐existing condition subsequent to COVID‐19 infection or vaccination. What's noteworthy is that all of these patients exhibited favorable responses when treated with anti‐IL‐17 or anti‐IL‐23 medications. Furthermore, the research findings unveiled substantially higher rates of clinical improvement at the 16‐week milestone when compared to the outcomes reported in both clinical trials and real‐world studies. These findings serve as compelling evidence supporting the therapeutic effectiveness of biologics in patients with relatively short disease durations and underscore the safety of these treatment approaches in the context of the ongoing COVID‐19 pandemic.^17^, ^22^, ^26^, ^27^, ^51^

Our analysis of ICU admission rate and the OR of COVID‐19 infection in psoriasis patients compared to the general population revealed significant heterogeneity between studies. Due to the limited number of studies available, further exploration of the sources of heterogeneity was challenging. To address this issue, we used a random effects model for our meta‐analysis and conducted subgroup analysis based on different countries. The COVID‐19 pandemic has affected various regions of the world at different times, resulting in variations in patient populations, phases of the pandemic, and healthcare systems. These factors may contribute to the observed heterogeneity in our results. It is worth noting that the limitations of our analysis include the limited number of studies available for inclusion and potential publication bias, which may affect the generalizability of our findings. Nevertheless, our study provides valuable insights into the association between psoriasis and COVID‐19 infection and highlights the need for further research to fully elucidate the relationship between the two conditions.

## 5 CONCLUSION

Several studies have investigated the relationship between psoriasis treatment and COVID‐19 infection. The data indicated that biologic therapy for psoriasis did not increase the risk of hospitalization due to COVID‐19 infection and may have even offered some protection against the onset and evolution of the infection. Additionally, treatment adherence was higher in psoriasis patients receiving biologic therapies than those receiving conventional therapies. These findings suggested that psoriasis treatment did not negatively impact COVID‐19 infection and that treatment could be continued on a case‐by‐case basis during the pandemic. Also, according to the conducted studies, the infection with the COVID‐19 virus itself does not affect the course of psoriasis, although the articles in this field could not be analyzed and more well‐designed studies are needed for certainty.

## AUTHOR CONTRIBUTIONS

A.G and N.A designed the research. S.F and F.S conducted the experiments. S.K wrote and F.S edited the paper. F.S and S.K performed the data analyses and A.G and N.G.A edited the manuscript. F.S, A.G, and N.G.A supervised the study. S.K editing grammatically, reviewing the methods and reanalyzing, and finalizing the revised paper. All authors have read and approved the content of the manuscript.

## CONFLICT OF INTEREST STATEMENT

The authors declare no conflict of interest.

## Supporting information

Supporting information.

## Data Availability

All data generated or analyzed during this study are included in this published article.
