# Supplementary material for: A systematic review and meta‐analysis of investigating the mutual impact of COVID‐19 and psoriasis: Focusing on COVID‐19 course in psoriasis and the opinion on biologics in this setting
Source: Immun Inflamm Dis. 2023 Nov 7;11(11):e1063. doi: 10.1002/iid3.1063 (PMC10629237; doi:10.1002/iid3.1063)
Supplement: Supplementary file 1 — Supporting information. [file IID3-11-e1063-s001.docx]

**Search Strategy**

| PubMed | ("Psoriasis"[Title] OR "Psoriatic"[Title] OR "pustulosis palmaris et plantaris"[Title] OR ("Pustulosis"[Title] AND "Soles"[Title]) OR "Psoriasis"[Title/Abstract] OR "Psoriasis"[MeSH Terms] OR "arthritis, psoriatic"[MeSH Terms]) AND ("COVID-19"[Title] AND "SARS-CoV-2"[Title/Abstract] AND "severe acute respiratory syndrome coronavirus 2"[Title/Abstract]) | 201 |
| --- | --- | --- |
| Web of knowledge (web of science) | (((TI=(Psoriasis )) OR TI=(Psoriatic)) OR TI=(Pustulosis Palmaris et Plantaris )) OR TI=(Pustulosis of Palms and Soles ) AND ( ((TI="COVID-19" OR TI= "SARS-CoV-2" OR TI= "severe acute respiratory syndrome coronavirus 2" ) ) | 242 |
| Google scholar | (("Psoriasis") OR "Psoriatic" OR " Pustulosis Palmaris et Plantaris " OR "Pustulosis of alms and Soles " ) AND ("covid-23" OR "severe acute respiratory syndrome coronavirus w" OR "sars-cov-w")) | 536 |
| Scopus | TITLE-ABS-KEY ( ( " Psoriasis " OR " Psoriatic " OR " Pustulosis Palmaris et Plantaris " OR "Pustulosis of Palms and Soles " ) ) AND ( "COVID-19" OR "SARS-CoV-2" OR "severe acute respiratory syndrome coronavirus 2" ) ) ) ) | 1001 |
